# Intermittent fasting improves metabolic outcomes in metabolic syndrome: a systematic review and meta-analysis with GRADE evaluation

**DOI:** 10.3389/fnut.2025.1664811

**Published:** 2025-12-11

**Authors:** Qian Song, Alaa Sultan H. Almutairi, Manal Fehaid A. Almutairi, Parmida Jamilian, Ahmed Abu-Zaid

**Affiliations:** 1 Department of Osteo-Internal Medicine, Tianjin Hospital, Tianjin University, Tianjin, China; 2 College of Medicine and Medical Sciences, Arabian Gulf University, Manama, Bahrain; 3 School of Pharmacy and Bio Engineering, Keele University, Keele, United Kingdom; 4 College of Medicine, Alfaisal University, Riyadh, Saudi Arabia

**Keywords:** intermittent fasting, metabolic syndrome, lipid profile, inflammatory, meta-analysis

## Abstract

**Background:**

Previous studies have demonstrated that intermittent fasting (IF) has garnered scientific attention and gained recognition for its beneficial effects on metabolic outcomes. However, the results are inconsistent. Accordingly, this systematic review and meta-analysis aimed to evaluate the effect of fasting on glycemic control, lipid profile, and inflammatory markers.

**Methods:**

Databases such as PubMed, Embase, Cochrane, Scopus, and Web of Science were used to retrieve relevant studies published until September 2025. The quality of the included studies was evaluated using the Cochrane Risk-of-Bias 2 (RoB2) tool. Moreover, the Grades of Recommendation, Assessment, Development, and Evaluation (GRADE) approach was employed to evaluate the quality of evidence.

**Results:**

A total of 10 studies, involving 701 individuals, were included in the current meta-analysis. The combined effect of various types of fasting significantly reduced fasting blood sugar (FBS) [standard mean difference (SMD) = −0.51; 95% confidence interval (CI): −0.81, −0.20; *p* = 0.001], insulin (SMD *=* −0.27; 95% CI: −0.52, −0.03; *p* = 0.027) and Homeostatic Model Assessment for Insulin Resistance (HOMA-IR) (SMD *=* −0.39; 95% CI: −0.65, −0.12; *p* = 0.004), and HbA1c (SMD = −0.25; 95% CI: −0.49, −0.02; *p* = 0.034) levels. Moreover, the regimen successfully exerted its beneficial effect on low-density lipoprotein cholesterol (LDL-C) (SMD = −0.34; 95% CI: −0.53, −0.14; *p* = 0.001) and interleukin-6 (IL-6) (SMD = −0.30; 95% CI: −0.57, −0.03; *p* = 0.029) levels as well. The sensitivity analysis indicated that excluding any single study had no effect on the overall effect size (ES) for FBS, blood sugar (BS), HOMA-IR, LDL-C, and high-density lipoprotein cholesterol (HDL-C). Moreover, the results of the GRADE approach scored high quality of evidence for FBS, insulin, HOMA-IR, HbA1c, total cholesterol (TC), LDL-C, and IL-6, which suggests the robustness of the results. No evidence of publication bias was detected using Egger’s and Begg’s test (*p* > 0.05).

**Conclusion:**

The findings suggest that intermittent fasting may have favorable effects on the metabolic panel, specifically, FBS, insulin, HOMA-IR, (HbA1c), LDL-C, and IL-6 levels.

## 1 Introduction

Metabolic health is influenced by glycemic levels and lipid profiles, both of which are associated with several diseases such as obesity, metabolic syndrome, impaired glucose tolerance, insulin resistance, and dyslipidemia (1–3). Moreover, low-grade inflammation triggers metabolic abnormalities, which subsequently affect metabolic health (4, 5). The growing global prevalence of these health conditions has raised public health concerns that need rapid intervention. In addition to pharmacological strategies, there is a need for the development of non-pharmacological therapies as an adjunctive and complementary therapy to enhance the success of metabolic interventions.

In this regard, fasting approaches are known to exert beneficial effects on metabolic health by modulating metabolic responses. Several types of fasting regimens have been developed with distinct metabolic effects. Intermittent fasting (IF) is a general and broad term representing dietary patterns alternating between periods of eating and fasting (6). Time-restricted fasting (TRF) limits food intake to a specific time frame within a day, whereas alternate-day fasting (ADF) alternates between days of normal eating and days of fasting (6–8). Evidence shows that a fasting regimen contributes to decreased fasting and postprandial glucose levels as well as improved insulin sensitivity, thereby serving as a key determinant of glycemic control (9). Furthermore, fasting regimens have been shown to increase lipolysis and decrease lipid synthesis, thereby improving dyslipidemia (10). In addition, several anti-inflammatory effects have been linked to fasting, although the extent of these effects may vary depending on the type of IF.

Although numerous studies have been conducted to evaluate the efficacy of various fasting regimens on metabolic health and generally reported beneficial outcomes, some studies have found no significant effects. Nonetheless, there is no consensus about the metabolic benefits of fasting. Therefore, this study aimed to provide a firm conclusion regarding the impact of fasting regimen on metabolic health.

## 2 Method

The study was conducted following the Cochrane Handbook for Systematic Reviews of Interventions (11) and the Preferred Reporting Items for Systematic Reviews and Meta-analyses (PRISMA) (12) guidelines. In addition, the study protocol was approved by the International Prospective Register of Systematic Reviews (CRD420251142741).

### 2.1 Search strategy

The PubMed, Scopus, Embase, Web of Science, and Cochrane Library were used to search for the relevant studies. The search was conducted from its inception through September 2025. Additionally, the reference list of relevant studies has been screened through a manual search. The search strategy was constructed based on an appropriate combination of Medical Subject Headings (MeSH) terms and keywords (Supplementary Table S1). The language was restricted to English.

### 2.2 Inclusion and exclusion criteria

Inclusion criteria for this study were defined using Population, Intervention, Comparison, and Outcome (PICO) criteria; Population (P): adults aged >18 years old with metabolic syndrome, Intervention (I): all types of fasting patterns, Comparison (C): placebo or control group, Outcome (O): blood glucose, glycemic control [fasting plasma glucose (FPG), HbA1c, Homeostatic Model Assessment for Insulin Resistance (HOMA-IR)], insulin, lipid profile [total cholesterol (TC), low-density lipoprotein cholesterol (LDL-c), high-density lipoprotein cholesterol (HDL-c), and triglyceride (TG)] levels, inflammatory markers [C-reactive protein (CRP), IL-6, and tumor necrosis factor (TNF-α)]. Studies with no placebo control group, studies with other designs (observational, animal studies), studies with duplicate data, and studies that evaluated the efficacy of Islamic fasting, which is structurally different from other types of fasting and complicates the comparison, were excluded.

#### 2.2.1 Data screening procedures and data extraction

All retrieved studies, duplicates, and references were managed using EndNote (version 9) to screen and remove any duplicates. Two independent researchers completed the screening process based on the title and abstract. Subsequently, the remaining studies were checked for their eligibility using full-texts. Any disagreements were resolved by consulting a third researcher. Similarly, the following data were extracted from the included studies: the name of first author, publication year, country, study design, gender, mean age, body mass index (BMI), and sample size of both intervention and control groups, study duration, health condition of study participants, type of fasting and control group, changes in main outcomes for both intervention and control groups [mean ± standard deviation (SD)].

### 2.3 Quality assessment and quality of evidence

The quality of included randomized controlled trials (RCTs) was evaluated using the RoB2 tool (13), which contains five domains: concealment of allocation, generation of random sequences, selective reporting, blinding of outcome assessment, and incomplete outcome data. Each of these domains was evaluated and scored as low, unclear, or high risk. In addition, the quality of evidence for all study outcomes was assessed using the Grades of Recommendation, Assessment, Development, and Evaluation (GRADE) guidelines (14). Accordingly, the quality of evidence was classified as high, moderate, and low for each outcome.

### 2.4 Statistical analysis

STATA Statistical Software version 14 (Stata Corp, College Station, TX, United States) was used to perform analyses using a random-effect model (15). The standard mean difference (SMD) and 95% confidence intervals (CIs) of changes for each outcome were used to express the overall effect size. The *I*^2^ index was used to present the heterogeneity of studies. In addition, a sensitivity analysis was conducted to evaluate the effect of individual studies on the overall effect size. Funnel plots and Begg’s and Egger’s tests were used to show any evidence of publication bias.

## 3 Results

### 3.1 Study selection and characteristics

**Figure 1:**
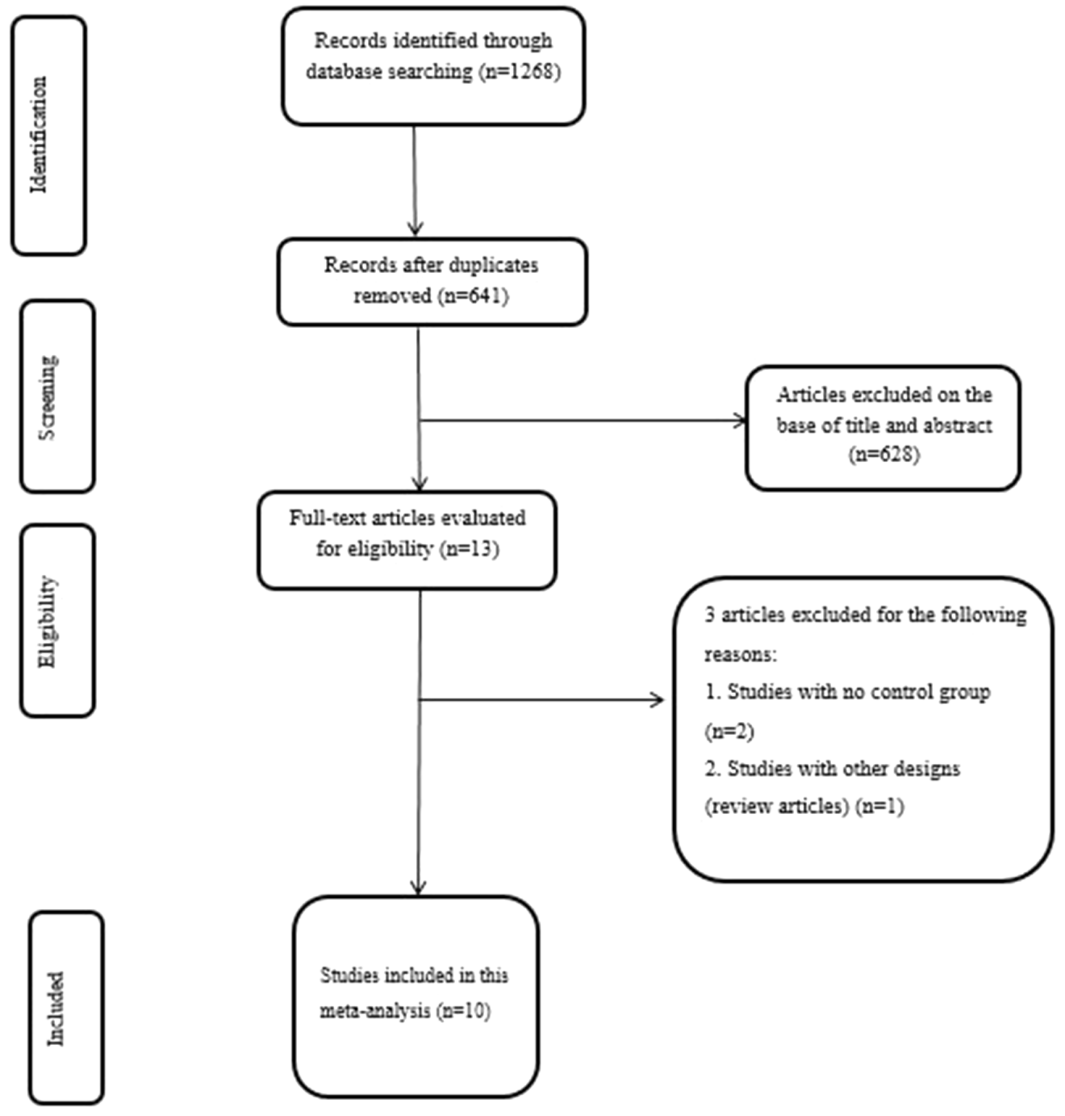
PRISMA flow chart of selection studies.

The search retrieved 1,268 records, of which 627 records were duplicates and were removed. The remaining 641 RCTs were checked by title and abstract, resulting in the exclusion of 628 irrelevant RCTs. Subsequently, 13 studies were screened using full-texts, and 3 articles that had no control group (*n* = 2) and a review (*n* = 1) were excluded. Finally, 10 studies were included for the meta-analysis. The PRISMA flow diagram is presented in Figure 1.

The study characteristics of the 10 studies included are presented in Table 1. The total sample size of the included RCTs comprised 701 individuals, with an age range of 25–75 years. The included trials were published between 2017 and 2025. Both men and women were included in all studies. BMI values indicated that all study subjects were classified as overweight or obese. The intervention duration ranged from 1 to 16 weeks. The majority of the participants were diagnosed with metabolic syndrome (MetS) or exhibited MetS components. Moreover, the type of intervention varied between studies, including TRF, ADF, and ICR.

**Table 1:** Basic characteristics of included RCTs.

Author, Year	Country	Study, Design	Gender	Mean age (Int, Cont)	BMI (Int, Cont)	N (Int, Cont)	Duration (week)	Health condition	Intervention type	Control group	Main outcome (Int)	Main outcome (Cont)
Sun et al. (2025) (22)	China	Parallel-arm, RCT	M/F	49.3 46.5	29 31.1	60 (30, 30)	12	MASLD	(Intermittent calorie restriction) Two successive days of fasting and 5 days of recovery per week. On fasting days: Consuming only a fixed amount of plant-based meal replacement, which provided 497.6 kcal/day	Traditional continuous calorie restriction	FBS (−12.6 ± 17.14) BS (−50.6 ± 90.2) HbA1c (−0.05 ± 0.54) HOMA-IR (−2.0 ± 3.34) Ins (−5.3 ± 9.63) TC (−3.9 ± 28.04) LDL (−4.6 ± 26.94) HDL (5.8 ± 5.86) TG (−24.8 ± 59.37)	FBS (−10.08 ± 14.78) BS (−71.6 ± 64.68) HbA1c (−0.1 ± 0.21) HOMA-IR (−1.6 ± 3.17) Ins (−3.7 ± 10.73) TC (−4.6 ± 23.71) LDL (0.8 ± 23.16) HDL (1.2 ± 5.42) TG (−45.1 ± 112.44)
Manoogian et al. (2024) (16)	USA	RCT	M/F	56.6 60.6	31.5	108 (54, 54)	12	MetS	(Time-restricted eating) A personalized 8–10-h eating window	-	FBS (−4.84 ± 6.22) HbA1c (−0.12 ± 0.20) HOMA-IR (−0.89 ± 2.18) Ins (−2.87 ± 8.72) LDL (−10.48 ± 25.78) HDL (−1.37 ± 9.34) TG (−7.77 ± 41.47) CRP (−0.19 ± 1.64)	FBS (−1.5 ± 7.39) HbA1c (−0.02 ± 0.17) HOMA-IR (−0.38 ± 1.63) Ins (−1.2 ± 5.97) LDL (−1.37 ± 24.98) HDL (−0.37 ± 9.82) TG (−13.94 ± 46.24) CRP (−0.09 ± 1.89)
Suthutvoravut et al. (2023) (17)	Thailand	RCT	M/F	55.5 55.2	–	46 (24, 22)	4	Patients with Impaired Fasting Glucose	(Time-restricted eating) Restriction of daily food intake to a 9 h window (between 8:00 a.m. and 5:00 p.m.), without any limitation on the types of food and beverages consumed	Usual care for impaired glucose	FBS (−3.03 ± 1.94) HbA1c (0.09 ± 0.08)	FBS (−1.79 ± 1.75) HbA1c (0.15 ± 0.08)
Cramer et al. (2022) (18)	Germany	SB, multicenter, parallel, RCT	M/F	58.6 ± 10.8 60.8 ± 10.8	33.7 ± 4.5	145 (73, 72)	10	MetS	(Intermittent calorie restriction) 2 calorie-restricted vegan days (max 1,200 kcal/day), followed by a 5-day modified fasting intervention (intake of 300–350 kcal/day, obtained from vegetable juices and vegetable broth)	Modified DASH diet, exercise, mindfulness	BS (−6.30 ± 11.77) HbA1c (−0.1 ± 0.31) HOMA-IR (−1.50 ± 1.55) Ins (−4.4 ± 4.4) TC (26.9 ± 30.94) LDL (−6.1 ± 24.70) HDL (−4.2 ± 9.63) TG (−71.6 ± 170.57) CRP (0.1 ± 0.25) IL-6 (−0.3 ± 1.62)	BS (4.30 ± 16.44) HbA1c (0.1 ± 0.44) HOMA-IR (−0.3 ± 1.48) Ins (−0.6 ± 4.6) TC (15.9 ± 30.33) LDL (2.7 ± 27.24) HDL (−2.5 ± 12.01) TG (−5.6 ± 66.81) CRP (0.1 ± 0.18) IL-6 (0.3 ± 1.68)
He et al. (2022) (21)	China	RCT	M/F	43 41.3	29.6 29.3	110 (55, 55)	12	MetS	(Time-restricted eating) The 8-h TRE group was instructed to consume all calories from 8 a.m. to 4 p.m. each day and fast from 4 p.m. to 8 a.m., or to consume all calories from 12 p.m. to 8 p.m. each day and fast from 8 p.m. to 12 p.m. (16-h fast). During the 8-h meal eating windows, they could eat ad libitum without any restriction on the quantities and types of food	Low-carbohydrate diet	FBS (−0.18 ± 0.48) HbA1c (0.0 ± 0.22) HOMA-IR (−1.04 ± 3.35) Ins (−3.3 ± 9.4) TC (0.03 ± 1.26) LDL (−4.6 ± 26.94) HDL (0.02 ± 0.22) TG (−0.03 ± 1.0)	FBS (0.07 ± 1.09) HbA1c (0.0 ± 0.22) HOMA-IR (−1.15 ± 2.21) Ins (−3.1 ± 7.7) TC (19.0 ± 0.88) LDL (0.8 ± 23.16) HDL (0.03 ± 0.22) TG (−0.15 ± 0.88)
Razavi et al. (2021) (35)	Iran	Single-center, RCT	M/F	41.3 43.1	31.3	69 (35, 34)	16	MetS	Alternate-day fasting diet	Calorie-restricted diet	CRP (−2.06 ± 1.18) IL-6 (−1.08 ± 2.7) TNF-*α* (−3.47 ± 5.77)	CRP (−0.97 ± 0.82) IL-6 (−0.61 ± 2.82) TNF-α (−2.21 ± 5.04)
Guo et al. (2021) (19)	China	RCT	M/F	40.2 ± 5.7 42.7 ± 4.1	28	39 (21, 18)	8	MetS	(Intermittent Calorie Restriction) 75% of energy restriction for 2 non-consecutive days a week and an ad libitum diet the other 5 days	Routine diet without dietary instructions	BS (−2.26 ± 5.40) HOMA-IR (−0.63 ± 0.93) Ins (−1.3 ± 5.0) TC (−1.6 ± 21.15) LDL (0.7 ± 16.90) HDL (−0.4 ± 5.82) TG (−35.4 ± 67.14)	BS (0.72 ± 12.31) HOMA-IR (0.12 ± 0.52) Ins (−0.07 ± 1.98) TC (−10.8 ± 20.35) LDL (20.9 ± 20.69) HDL (8.1 ± 6.83) TG (10.6 ± 80.70)
Kunduraci and Ozbek (2020) (33)	Turkey	RCT	M/F	47.44 ± 2.17 48.76 ± 2.13	36.58 ± 0.93	65 (32, 33)	12	MetS	(Intermittent calorie restriction)	Continuous energy restriction (CER)	BS (−15.47 ± 5.70) HbA1c (−0.32 ± 0.18) HOMA-IR(−1.29 ± 0.45) Ins (−2.23 ± 1.64) TC (−29.32 ± 4.88) LDL (−17.0 ± 3.57) HDL (0.53 ± 1.12) TG (−41.84 ± 15.42)	BS (−13.12 ± 4.29) HbA1c (−0.31 ± 0.15) HOMA-IR (−0.94 ± 0.49) Ins (−2.39 ± 1.31) TC (−29.36 ± 5.25) LDL (−15.97 ± 3.49) HDL (−0.38 ± 1.37) TG (−40.0 ± 20.77)
Parvaresh et al. (2019) (34)	Iran	RCT	M/F	44.6 46.4	31.1 ± 3.35	69 (35, 34)	8	MetS	(Modified Alternate-Day Fasting) A very low-calorie diet (75% energy restriction) during the 3 fast days (Saturday, Monday, Wednesday) and then ate a diet that provided 100% of their energy needs on each feed day (Sunday, Tuesday, Thursday)	Calorie restriction	FBS (−6.00 ± 5.78) HOMA-IR (−2.42 ± 3.82) TC (−11.0 ± 21.99) LDL (−6.0 ± 17.83) HDL (−1.0 ± 5.56) TG (−52.0 ± 67.25)	FBS (0.0 ± 5.34) HOMA-IR (−1.57 ± 4.09) TC (−9.0 ± 22.57) LDL (0.0 ± 17.89) HDL (−1.0 ± 5.97) TG (−40.0 ± 69.85)
Li et al. (2017) (20)	Germany	RCT	M/F	25–75	–	32 (16, 16)	1	T2DM	(Intermittent calorie restriction) 2 pre-fasting days with moderate caloric restriction, followed by 7 modified fasting days	–	BS (−10.70 ± 17.55) HbA1c (−2.2 ± 12.0) HOMA-IR(−1.50 ± 4.6) Ins (−3.4 ± 6.78) TC (−0.5 ± 27.1) LDL (−2.6 ± 26.9) HDL (6.5 ± 23.3) TG (−26.6 ± 88.5)	BS (−38.5 ± 27.18) HbA1c (−2.2 ± 8.7) HOMA-IR(−1.50 ± 2.1) Ins (−0.2 ± 7.22) TC (−15.5 ± 27.4) LDL (−7.8 ± 17.3) HDL (−2.3 ± 6.9) TG (−2.5 ± 81.9)

RCT, randomized clinical trial; Int, Intervention; Cont, Control; M, Male; F, Female; MASLD, Metabolic dysfunction-associated steatotic liver disease; MetS, Metabolic syndrome; FBS, Fasting blood sugar; BS, Blood sugar; HOMA-IR, Homeostasis model assessment-estimated insulin resistance; TC, Total cholesterol; LDL, Low-density lipoprotein; HDL, High-density lipoprotein; TG, Triglyceride; TRE, Time-restricted eating.

### 3.2 The impact of the fasting approach on glycemic control

**Figure 2:**
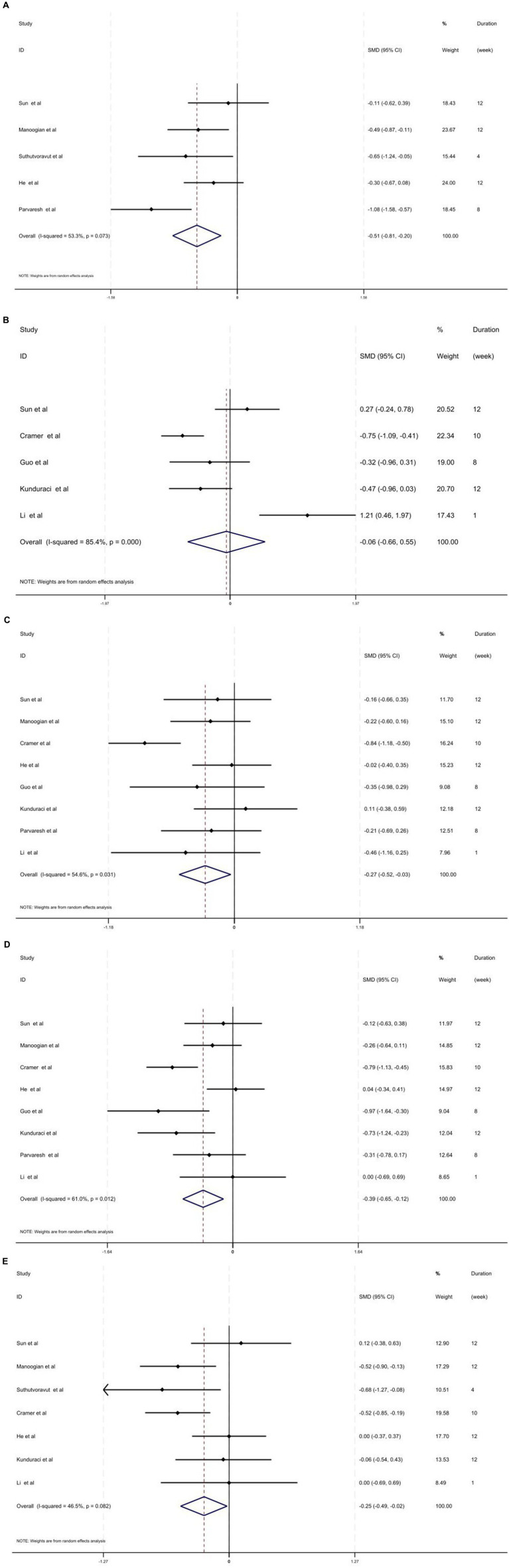
**(A)** Forest plot of intermittent fasting vs. control on FBS level. **(B)** Forest plot of intermittent fasting vs. control on the BS level. **(C)** Forest plot of intermittent fasting vs. control on insulin level. **(D)** Forest plot of intermittent fasting vs. control on HOMA-IR level. **(E)** Forest plot of intermittent fasting vs. control on HbA1c level.

The pooled analysis of five studies encompassing 393 individuals evaluating the effect of fasting regimen on fasting blood sugar (FBS) indicated a significant reduction (SMD = −0.51; 95% CI: −0.81, −0.20, *p =* 0.001; *I*^2^ = 53.3%, *p* = 0.073) (Figure 2A). The subgroup analysis revealed that the fasting approach has a significant reducing effect on older adults (>50 years old) with a higher BMI (>30 kg/m^2^) and in short-term treatment (*p* < 0.05). In addition, it has demonstrated that most of this beneficial effect is related to TRF treatment rather than other fasting methods (*p* < 0.05) (Table 2).

**Table 2:** Pooled estimate effects of fasting regimen on the metabolic markers across different subgroups.

Group	Number of comparisons	ES (95% CI)	*p*-value	*I*^2^ (%)	*p*-heterogeneity
FBS					
Age					
<50 years	3	−0.49 (−1.03, 0.05)	0.077	75.7%	0.016
>50 years	2	−0.54 (−0.86, −0.21)	0.001	0.0%	0.661
BMI					
<30	2	−0.23 (−0.53, 0.07)	0.133	0%	0.567
>30	2	−0.76 (−1.33, −0.18)	0.010	69.6%	0.070
NR	1	−0.65 (−1.24, −0.05)	0.033	–	–
Duration					
<8	2	−0.89 (−1.31, −0.47)	<0.001	14.0%	0.281
>8	3	−0.33 (−0.57, −0.09)	0.006	0.0%	0.497
Type of fasting					
TRF	3	−0.43 (−0.68, −0.19)	<0.001	0.0%	0.582
ADF	1	−1.06 (−1.58, −0.57)	<0.001	–	–
ICR	1	−0.11 (−0.62, 0.39)	0.663	–	–
Sample size					
<100	3	−0.61 (−1.19, −0.03)	0.038	71.4%	0.030
>100	2	−0.39 (−0.66, −0.12)	0.004	0.0%	0.484
BS					
Age					
<50 years	3	−0.17 (−0.63, 0.30)	0.484	55.1%	0.108
>50 years	2	0.20 (−1.72, 2.13)	0.838	95.4%	<0.001
BMI					
<30	2	0.0 (−0.57, 0.58)	0.988	50.6%	0.155
>30	2	−0.66 (−0.94, −0.38)	<0.001	0.0%	0.350
NR	1	1.21 (0.46, 1.97)	0.002	–	–
Duration					
<8	2	0.43 (−1.07, 1.94)	0.574	89.2%	0.002
>8	3	−0.34 (−0.93, 0.25)	0.263	81.3%	0.005
Insulin					
Age					
<50 years	5	−0.10 (−0.31, 0.12)	0.371	0.0%	0.786
>50 years	3	−0.52 (−0.96, −0.09)	0.019	65.5%	0.055
BMI					
<30	3	−0.12 (−0.39, 0.15)	0.381	0.0%	0.677
>30	4	−032 (−0.73, 0.10)	0.137	75.2%	0.007
NR	1	−0.46 (−1.16, 0.25)	0.202	–	–
Duration					
<8	3	−0.31 (−0.64, 0.03)	0.072	0.0%	0.845
>8	5	−0.25 (−0.60, 0.11)	0.175	73.5%	0.005
Type of fasting					
TRF	2	−0.12 (−0.39, 0.14)	0.368	0.0%	0.461
ADF	1	−0.21 (−0.69, 0.26)	0.374	–	–
ICR	5	−0.36 (−0.75, 0.03)	0.073	65.4%	0.021
Sample size					
<100	5	−0.17 (−0.41, 0.07)	0.168	0.0%	0.693
>100	3	−0.37 (−0.87, 0.13)	0.147	82.3%	0.004
HOMA-IR					
Age					
<50	5	−0.37 (−0.73, −0.02)	0.039	61.4%	0.035
>50	3	−0.41 (−0.86, 0.04)	0.076	68.1%	0.043
BMI					
<30	3	−0.29 (−0.83, 0.24)	0.284	70.4%	0.034
>30	4	−0.53 (−0.81, −0.24)	<0.001	46.5%	0.132
NR	1	0.0(−0.69, 0.69)	1.00	–	–
Duration					
<8	3	−0.42 (−0.93, 0.09)	0.107	52.7%	0.121
>8	5	−0.38 (−0.72, −0.03)	0.031	70.8%	0.008
Type of fasting					
TRF	2	−0.11 (−0.41, 0.19)	0.463	20.0%	0.264
ADF	1	−0.31 (−0.78, 0.17)	0.205	–	–
ICR	5	−0.55 (−0.90, −0.20)	0.002	55.3%	0.063
Sample size					
<100	5	−0.42 (−0.75, −0.09)	0.013	43.4%	0.132
>100	3	−0.34 (−0.83, 0.14)	0.166	81.4%	0.005
HbA1c					
Age					
<50	3	0.01 (−0.24, 0.27)	0.909	0.0%	0.786
>50	4	−0.49 (−0.71, −0.27)	<0.001	0.0%	0.502
BMI					
<30	2	0.04 (−0.26, 0.34)	0.779	0.0%	0.704
>30	3	−0.41 (−0.67, −0.15)	0.002	26.6%	0.256
NR	2	−0.36 (−1.03, 0.30)	0.283	52.7%	0.146
Duration					
<8	2	−0.36 (−1.03, 0.30)	0.283	52.7%	0.146
>8	5	−0.22 (−0.50, 0.05)	0.104	54.6%	0.066
Type of fasting					
TRF	3	−0.36 (−0.77, 0.05)	0.082	61.7%	0.074
ICR	4	−0.17 (−0.50, 0.17)	0.324	47.5%	0.126
Sample size					
<100	4	−0.14 (−0.48, 0.20)	0.425	32.1%	0.220
>100	3	−0.35 (−0.68, −0.02)	0.040	60.9%	0.078

However, the combined effect of studies which have evaluated the effects of fasting regimen on BS illustrated a non-significant effect (SMD = −0.06; 95% CI: −0.66, 0.55, *p =* 0.849; *I*^2^ = 85.4%, *p* < 0.001) (Figure 2B). Similarly, subgroup analysis showed that individuals with higher BMI had reduced BS levels following a fasting regimen (*p* < 0.05) (Table 2).

In total, eight studies including 628 individuals assessed the effect of fasting on insulin levels. It has been demonstrated that a fasting regimen could have a significant and beneficial reducing effect on insulin levels (SMD = −0.27; 95% CI: −0.52, −0.03; *p* = 0.027) with moderate heterogeneity (*I*^2^ = 54.6.0%, *p* = 0.031) (Figure 2C). The subgroup analysis revealed that older adults (>50 years old) may benefit more from the fasting approach in terms of insulin levels (*p* < 0.05) (Table 2).

Overall, eight trials with a total of 628 adults indicated that a fasting regimen could reduce HOMA-IR levels significantly (SMD = −0.39; 95% CI: −0.65, −0.12, *p =* 0.004; *I*^2^ = 61.0%, *p* = 0.012) (Figure 2D). The subgroup analysis showed that the fasting regimen has a more favorable effect on younger adults (<50 years old) and obese individuals (BMI > 30 kg/m^2^) (*p* < 0.05). In addition, long-term fasting (>8 weeks) was associated with a significant reduction in HOMA-IR levels as well (*p* < 0.05). Moreover, the subgroup analysis based on the type of fasting method revealed a significant reduction effect for the ICR methodology (*p* < 0.05) (Table 2).

In addition, the effect of fasting on HbA1c levels was explored from 7 studies with 566 participants. Random-effects model indicated that fasting significantly reduced HbA1c levels (SMD = −0.25; 95% CI: −0.49; −0.02, *p =* 0.034; *I^2^* = 46.5%, *p* = 0.082) (Figure 2E). The results of the subgroup analysis revealed that older adults (>50 years old) with higher BMI (>30 kg/m^2^) demonstrated significantly greater improvements than other subgroups (Table 2).

### 3.3 The impact of the fasting approach on lipid profile

**Figure 3:**
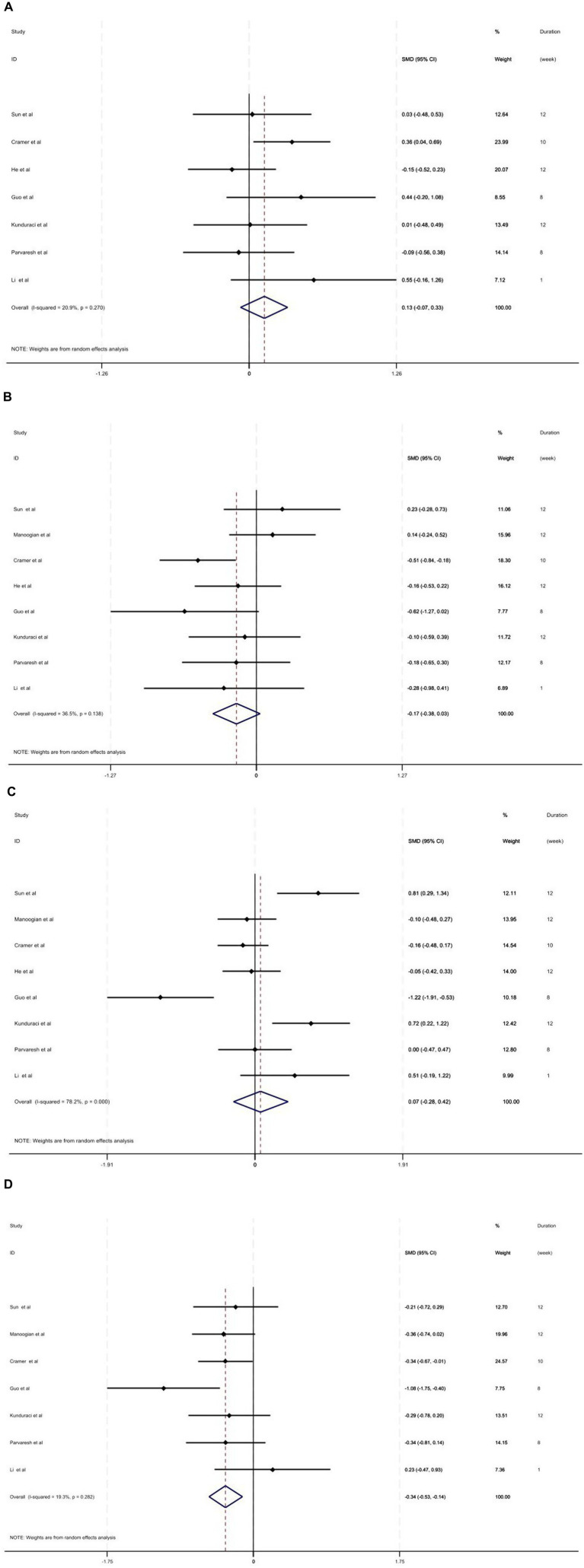
**(A)** Forest plot of intermittent fasting vs. control on TC level. **(B)** Forest plot of intermittent fasting vs. control on TG level. **(C)** Forest plot of intermittent fasting vs. control on HDL-C level. **(D)** Forest plot of intermittent fasting vs. control on LDL-C level.

Seven studies, including 520 individuals, investigated the effect of fasting regimen on TC levels. Accordingly, it has been shown that fasting did not significantly affect the TC level (SMD = 0.13; 95% CI: −0.07, 0.33, *p =* 0.212; *I^2^* = 20.9%, *p* = 0.270) (Figure 3A). Fasting intervention showed similar effects in terms of TG (SMD = −0.17; 95% CI: −0.38, 0.03, *p =* 0.097; *I^2^* = 36.5%, *p* = 0.138) (Figure 3B) and HDL-C (SMD = 0.07; 95% CI: −0.28, 0.42, *p =* 0.690; *I^2^* = 78.2%, *p* < 0.001) (Figure 3C) levels. However, the pooled effect of seven studies (518 participants) showed a reduced effect on LDL-C levels following fasting (SMD = −0.34; 95% CI: −0.53, −0.14, *p =* 0.001; *I^2^* = 19.3%, *p* = 0.282) (Figure 3D). In addition, heterogeneity for TC, TG, and LDL-C levels was below 50%, and therefore, no subgroup analysis was performed. However, the subgroup analysis carried out for HDL-C failed to identify the source of heterogeneity in the HDL-C values (Table 2).

### 3.4 The impact of the fasting approach on inflammatory markers

**Figure 4:**
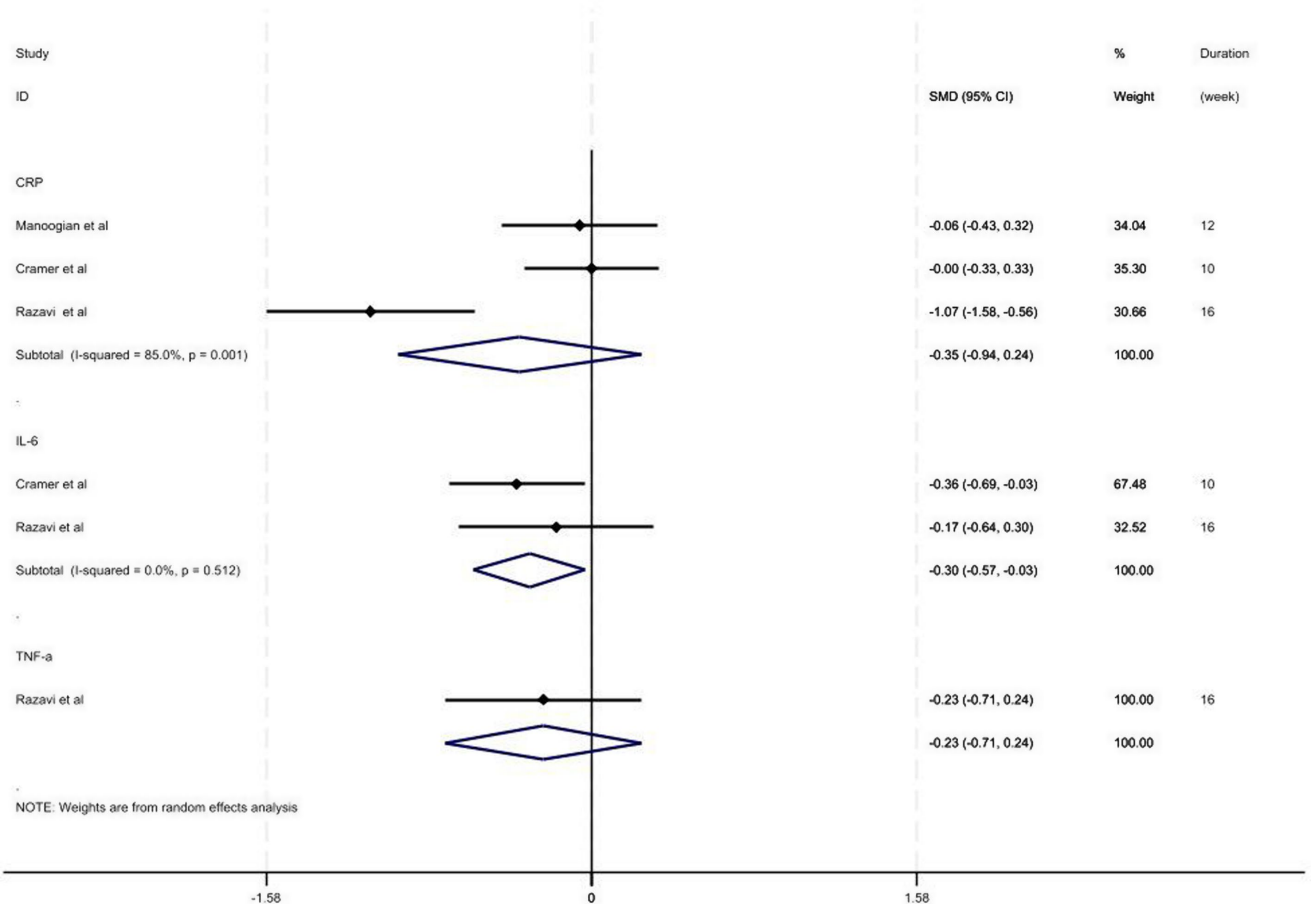
Forest plot of intermittent fasting vs. control on inflammatory markers.

The present study also attempted to evaluate the effect of fasting on inflammatory markers (CRP, IL-6, and TNF-*α*). The meta-analysis showed that fasting regimen has significant ameliorative effects on the IL-6 levels (SMD = −0.30; 95% CI: −0.57, −0.03, *p =* 0.029; *I^2^* = 0.0%, *p* = 0.0512) (Figure 4). However, it had no significant effect on CRP and TNF-α levels (*p* > 0.05).

### 3.5 Sensitivity analyses and publication bias

Sensitivity analyses employed a leave-one-out approach to exclude an individual study and evaluate the overall effect on the results. It was shown that excluding each individual study has no effect on the overall results of FBS, BS, and HOMA-IR. In contrast, the studies by Manoogian et al. (16), Suthutvoravut et al. (17), and Cramer et al. (18) have the potential to alter the levels of HbA1c to a non-significant form. Additionally, sensitivity analyses showed that the exclusion of Manoogian et al. (16), Cramer et al. (18), Guo et al. (19), and Li et al. (20) altered the pooled results for insulin levels. In addition, sensitivity analyses of lipid markers demonstrated that excluding the study by He et al. (21) could affect the overall results of TC. Similarly, excluding Sun et al.(22), Manoogian et al. (16) were able to significantly alter the effect of fasting on TG levels. Nonetheless, the leave-one-out approach had no significant effect on LDL-C and HDL-C outcomes.

Publication bias for included studies was assessed using Egger’s and Begg’s tests. No evidence of publication bias was observed for any glycemic-related outcomes (Egger’s and Begg’s test results: FBS, 0.551 and 0.806; BS, 0.087 and 0.086; insulin, 0.539 and 0.711; HOMA-IR, 0.994 and 0.902; and HbA1c, 0.510 and 0.764). Similarly, no publication bias was detected for lipid markers (TC, 0.667 and 0.230; LDL-C, 0.942 and 0.230; HDL-C, 0.718 and 0.266; and TG, 0.954 and 1.0). Visual inspection of the funnel plots also indicated no publication bias across all study outcomes as presented in Supplementary Figure S1.

### 3.6 Quality assessment and GRADE approach

Quality assessment of the included studies using the RoB2 tool classified three studies as having a low risk of bias, while four studies had some concerns. All of the included studies had a low risk of bias for the following domains: randomization process, selection of the reported result, and missing outcome data. The details of the quality assessment based on the domains are presented in Table 3.

**Table 3:** Results of risk of bias assessment for included RCTs in the present study.

Author, Year	Randomization process	Deviation from intended interventions	Selection of the reported result	Measurement of the outcome	Missing outcome data	General risk of bias
Sun et al. (2025) (22)	Low	High	Low	Low	Low	High
Manoogian et al. (2024) (16)	Low	Unclear	Low	Unclear	Low	Some concern
Suthutvoravut et al. (2023) (17)	Low	Low	Low	Low	Low	Low
Cramer et al. (2022) (18)	Low	Unclear	Low	Low	Low	Some concern
Sun et al. (2025) (22)	Low	Unclear	Low	Low	Low	Some concern
Guo et al. (2021) (19)	Low	High	Low	High	Low	High
Razavi et al. (2021) (35)	Low	Low	Low	Low	Low	Low
Kunduraci and Ozbek (2020) (33)	Low	Unclear	Low	Unclear	Low	Some concern
Parvaresh et al. (2019) (34)	Low	Low	Low	Low	Low	Low
Li et al. (2017) (20)	Low	High	Low	Low	Low	High

Each study was assessed for risk of bias using the ROB2 tool. Each domain was rated as “high risk” if it contained methodological issues that may have affected the results, “low risk” if the flaw was deemed inconsequential, and “some concern” if information was insufficient to determine.

The GRADE quality of evidence for the effect of fasting on FBS, insulin, HOMA-IR, HbA1c, TC, LDL-C, and IL-6 levels was assessed as high, suggesting the robustness of the results. However, the effect of fasting was considered moderate for TG and TNF-*α* values (Table 4).

**Table 4:** Summary of findings and quality of evidence assessment using the GRADE approach.

Outcomes	No of patients (meta-analysis)	SMD (95% CI)	Risk of bias	Inconsistency	Indirectness	Imprecision	Publication bias	Quality of evidence
BS (mg/dL)	341 (5)	−0.06 (−0.66, 0.55)	Not serious	Serious	Not serious	Serious	Not serious	Low
FBS (mg/dL)	393 (5)	−0.51 (−0.81, −0.20)	Not serious	Not serious	Not serious	Not serious	Not serious	High
Insulin (mU/L)	628 (8)	−0.27 (−0.52, −0.03)	Not serious	Not serious	Not serious	Not serious	Not serious	High
HOMA-IR	628 (8)	−0.39 (−0.65, −0.12)	Not serious	Not serious	Not serious	Not serious	Not serious	High
HbA1c	566 (7)	−0.25 (−0.49, −0.02)	Not serious	Not serious	Not serious	Not serious	Not serious	High
TC (mg/dL)	520 (7)	0.13 (−0.07, 0.33)	Not serious	Not serious	Not serious	Serious	Not serious	High
LDL-C (mg/dL)	261 (7)	−0.34 (−0.53, −0.14)	Not serious	Not serious	Not serious	Not serious	Not serious	High
HDL-C (mg/dL)	628 (8)	0.07 (−0.28, 0.42)	Not serious	Serious	Not serious	Serious	Not serious	Low
TG (mg/dL)	628 (8)	−0.17 (−0.38, 0.03)	Not serious	Not serious	Not serious	Serious	Not serious	Moderate
CRP	322 (3)	−0.35 (−0.94, 0.24)	Not serious	Serious	Not serious	Serious	Not serious	Low
IL-6	214 (2)	−0.30 (−0.57, −0.03)	Not serious	Not serious	Not serious	Not serious	Not serious	High
TNF-α	69 (1)	−0.23 (−0.71, 0.24)	Not serious	Not serious	Not serious	Serious	Not serious	Moderate

## 4 Discussion

This systematic review and meta-analysis of 10 RCTs provides a valuable insight regarding the impact of IF on metabolic health. It has been demonstrated that IF is associated with an improvement in glycemic control and insulin sensitivity, as evidenced by a reduction in FBS, insulin, HOMA-IR, and HbA1c levels. These favorable effects seem to be mediated through several probable mechanisms. Previous studies indicate that IF inhibits gluconeogenesis in hepatocytes, thereby reducing HOMA-IR levels and improving overall insulin sensitivity (23). In addition, IF has been reported to promote pancreatic islet neogenesis, which may enhance the *β*-cell function and improve glycemic regulation (23, 24). It is worth noting that postabsorptive glucose levels are dependent on daily intake and dietary fluctuations and appear to be less influenced by IF intervention. Similarly, the higher sensitivity and responsiveness of postprandial glucose to short-term interventions cannot be ignored. In contrast, FBS, after a night of fasting, and HbA1c levels are more stable and provide a precise judgment in this regard (25). Another probable explanation that can unveil these findings is attributed to the way that fasting exerts its effect. The fasting regimen predominantly influences improvements in hepatic glucose production and insulin sensitivity, rather than postprandial glucose levels. In addition, subgroup analysis indicated that fasting contributes to a more significant improvement in FBS, BS, HOMA-IR, and HbA1c levels in obese individuals (BMI > 30 kg/m^2^). These findings are promising for individuals with obesity who are at risk of MetS and diabetes progression. It could be a beneficial strategy for the management of metabolic health in high-risk populations.

Additionally, subgroup analyses demonstrated that fasting intervention resulted in a greater reduction in FBS, HOMA-IR, and HbA1c levels among participants aged **>**50 years. Some probable mechanisms may explain this age-specific finding. Older adults tended to show higher baseline FBS, insulin, and HbA1c values, and the aging process is accompanied by insulin resistance. Likewise, they may respond more efficiently to fasting intervention.

In addition, fasting was accompanied by a significant reduction in LDL-C levels as well. This literature review has illustrated that fasting suppresses sterol regulatory element-binding protein 2 (SREBP-2), thereby inhibiting the activation of 3-hydroxy-3-methylglutaryl-CoA (HMG-CoA) synthase and ultimately reducing the synthesis of TC (26–28). Moreover, IF induces proliferator-activated receptor alpha (PPAR*α*), which participates in the activation of the JMJD3-SIRT1-PPAR α complex (26, 29). Through histone modification, this pathway promotes *β*-oxidation of fatty acids (26). Although improvements in TC, TG, and HDL-C levels might be expected with IF, these lipid markers are influenced by multiple factors such as genetic variability, dietary composition, and lifestyle habits, which may mask the overall pooled effect in meta-analyses (30).

Furthermore, IF was associated with an improvement in IL-6 levels, suggesting an anti-inflammatory effect of IF (31, 32). However, there are only a few number of studies that evaluate other inflammatory markers, which limits the statistical power to assess their actual effect.

From a clinical perspective, the effect sizes (ESs) of the glycemic markers were all below the minimal clinically important difference (MCID), indicating that IF may have only a limited clinical effect, despite achieving statistical significance. The pooled ESs for HbA1c (SMD: −0.25) are below the MCID (MCID: 0.3–0.5), suggesting that although the reduction in HbA1c levels was statistically significant, it is unlikely to be clinically meaningful too. Similarly, the effect sizes of other evaluated markers were below the MCID. These findings highlight the need for further studies to determine whether IF can serve as an effective adjunctive strategy to improve glycemic control in high-risk populations. In addition, the majority of the included RCTs were of short duration (≤16 weeks), which limits the ability to assess the long-term efficacy.

Overall, the quality assessment of the included studies revealed a low publication bias for the majority of the included studies. In addition, the certainty of the evidence, as assessed using the GRADE method, showed a higher level of certainty for the HOMA-IR outcome, suggesting that these findings are more reliable and generalizable. Additionally, HbA1c, insulin, TC, TG, LD-C, and HDL-C levels received moderate certainty of evidence, reflecting a reasonable but less robust level of confidence. The current study also had some limitations that need to be addressed. The type of fasting regimen may affect the observed outcomes.

## 5 Conclusion

In conclusion, the current systematic review and meta-analysis demonstrate that fasting regimens may help improve glycemic control by significantly reducing the FBS, HbA1c, and HOMA-IR levels. Similarly, IF was also successful in reducing the LDL-C levels.

## Data Availability

The datasets presented in this study can be found in online repositories. The names of the repository/repositories and accession number(s) can be found in the article/Supplementary material.
