# Supplementary material for: Intermittent fasting improves metabolic outcomes in metabolic syndrome: a systematic review and meta-analysis with GRADE evaluation
Source: Front Nutr. 2025 Dec 11;12:1664811. doi: 10.3389/fnut.2025.1664811 (PMC12738305; doi:10.3389/fnut.2025.1664811)
Supplement: Supplementary file 1 [file Supplementary_file_1.docx]

**Supplementary File**

Supplementary Table 1.

("Fasting"[Mesh] OR "Fasting"[tiab] OR "intermittent fasting"[tiab] OR "intermittent‑fasting"[tiab] OR "time restricted feeding"[tiab] OR "time‑restricted feeding"[tiab] OR "time restricted eating"[tiab] OR "time‑restricted eating"[tiab] OR "time restricted"[tiab] OR "alternate‑day fasting"[tiab] OR "alternate day fasting"[tiab] OR "ADF"[tiab] OR "periodic fasting"[tiab] OR "prolonged fasting"[tiab] OR "Ramadan"[tiab] OR "religious fasting"[tiab] OR "fasting‑mimicking"[tiab] OR "fasting mimicking"[tiab] OR "fasting mimicking diet"[tiab] OR "caloric restriction"[Mesh] OR "caloric restriction"[tiab] OR "energy restriction"[tiab] OR fast*[tiab]) AND ("Metabolic Syndrome"[Mesh] OR "metabolic syndrome"[tiab] OR "Syndrome X"[tiab] OR "Impaired Glucose Tolerance"[Mesh] OR "impaired glucose tolerance"[tiab] OR prediabetes[Mesh] OR prediabetes[tiab] OR "impaired fasting glucose"[tiab] OR IFG[tiab] OR "insulin resistance"[Mesh] OR "insulin resistance"[tiab]) AND ("randomized controlled trial"[Publication Type] OR "controlled clinical trial"[Publication Type] OR "controlled clinical trial"[All Fields] OR randomized[Title/Abstract] OR randomized [Title/Abstract] OR placebo[Title/Abstract] OR "clinical trials as topic"[MeSH Terms] OR "cross-over studies"[MeSH Terms] OR "cross-over studies"[All Fields] OR "cross over studies"[All Fields] OR "Cross-over study"[All Fields] OR "Cross over study"[All Fields] OR "clinical trial"[Publication Type] NOT animals[All Fields] ).


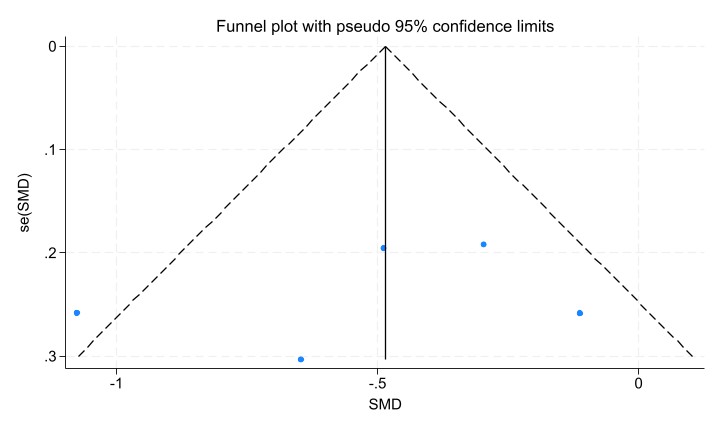


**Supplementary Fig. 1**. The funnel plot of FBS.


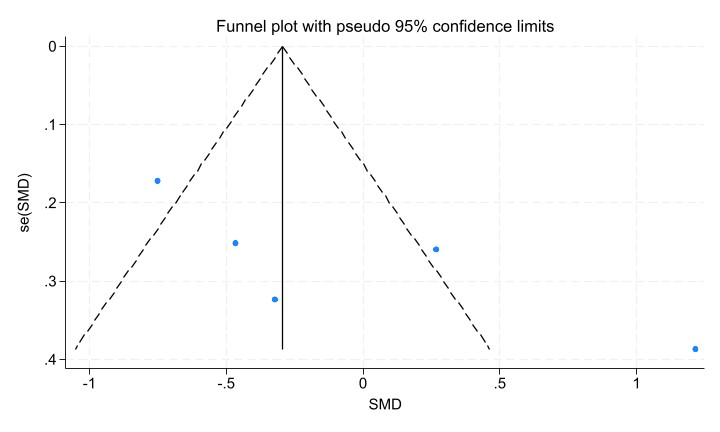


**Supplementary Fig. 2**. The funnel plot of BS.


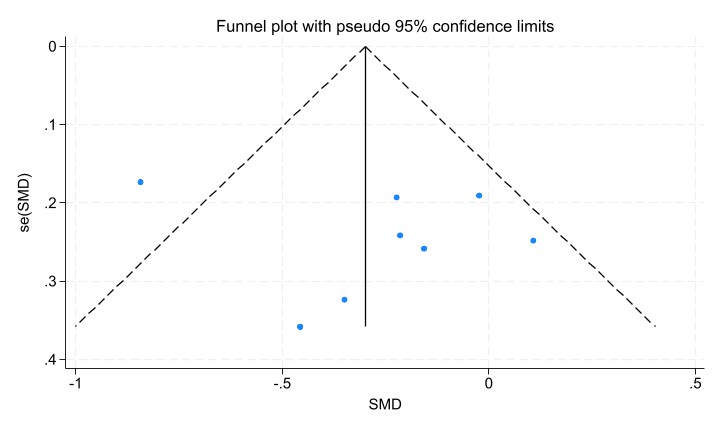


**Supplementary Fig. 3**. The funnel plot of insulin.


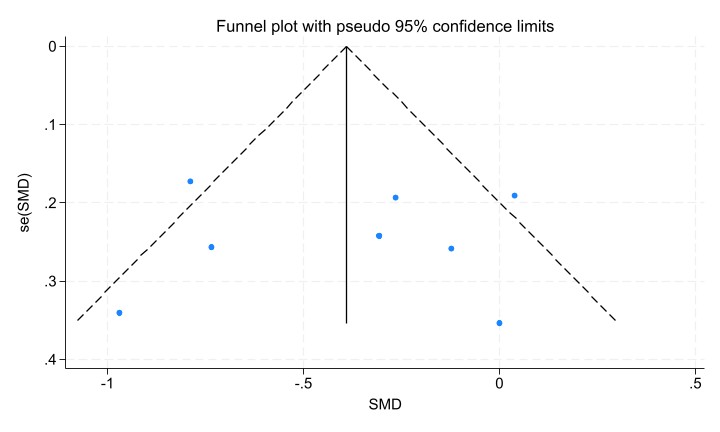


**Supplementary Fig. 4**. The funnel plot of HOMA-IR.


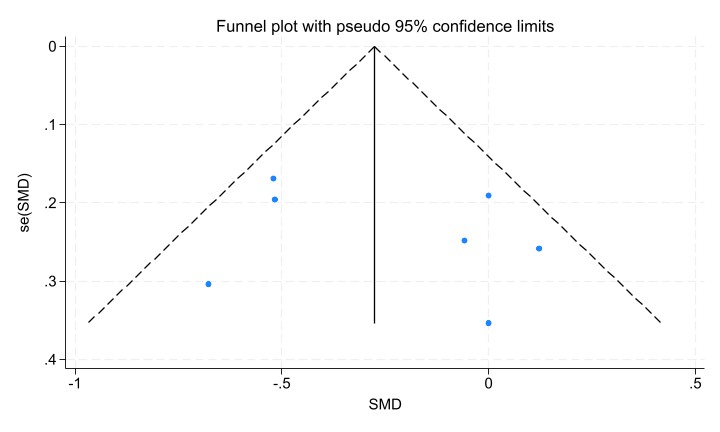


**Supplementary Fig. 5**. The funnel plot of HbA1c.


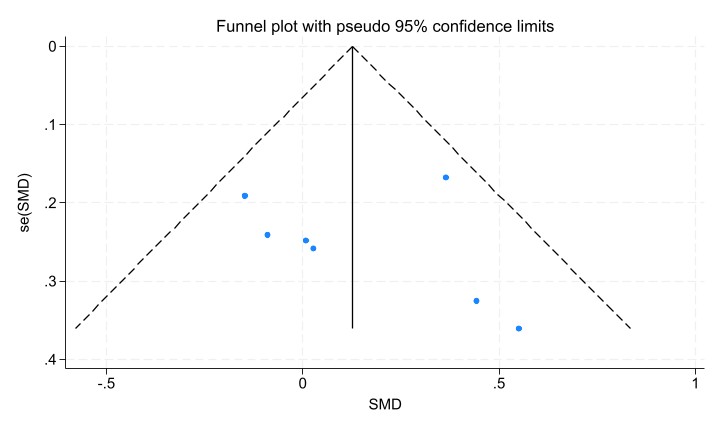


**Supplementary Fig. 6**. The funnel plot of TC.


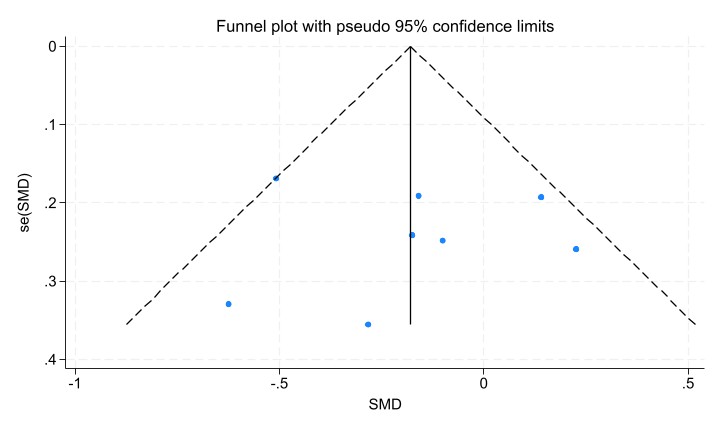


**Supplementary Fig. 7**. The funnel plot of TG.


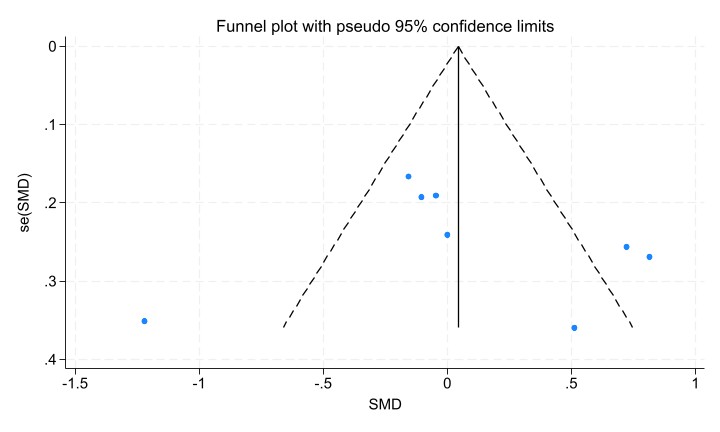


**Supplementary Fig. 8**. The funnel plot of HDL-C


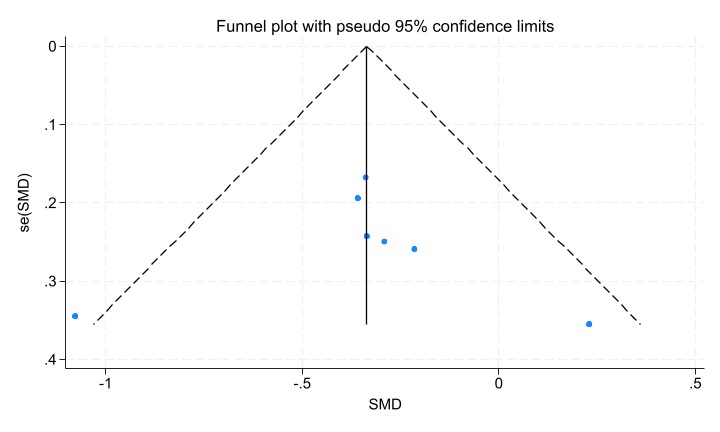


**Supplementary Fig. 9**. The funnel plot of LDL-C.
